# Compressive strength evaluation of two composites with and without fiber reinforcement used as restorative material in incisal edge: An *In-vitro* study

**DOI:** 10.4317/jced.62410

**Published:** 2025-02-01

**Authors:** P V S Tejaswini Yadav Sagala, Sayesh Vemuri, Anila Bandlapally Sreenivasa Guptha, Roopadevi Garlapati, Tsalla Krishna Ravali, Maddarapu Vamsi Krishna

**Affiliations:** 1 Post Graduate, Department of Conservative Dentistry & Endodontics, Sibar Institute of Dental Sciences, Takkellapadu, Andhra Pradesh, India; 2 Professor & HOD, Department of Conservative Dentistry & Endodontics, Sibar Institute of Dental Sciences, Takkellapadu, Andhra Pradesh, India; 3 Professor, Department of Conservative Dentistry & Endodontics, Sibar Institute of Dental Sciences, Takkellapadu, Andhra Pradesh, India

## Abstract

**Background:**

To evaluate compressive strength of two composites with and without fiber reinforcement used as restorative material in incisal edge.

**Material and Methods:**

Sixty extracted human maxillary incisors were collected and divided into four groups (n=15). In group 1and 3: fracture line is beveled circumferentially, etched with 37% phosphoric acid gel for 15 seconds, bonding agent applied, and the lost tooth structure was incrementally built with nano-composites. In group 2 and 4: grooves are prepared for placing fibers. Fracture line is beveled circumferentially, fiber is placed into the flowable composite in the prepared groove area and remaining portion built incrementally with nano-composite. All samples were subjected to universal testing machine to evaluate compressive strength and observed in stereomicroscope to analyze mode of failure. The obtained data is analyzed using one-way ANOVA and Tukey’s Post-hoc tests.

**Results:**

Group 2 and 4 showed significantly higher fracture resistance. No significant difference is observed between group 1 and 3, group 2 and 4. More mixed failure were seen in group 1 and 3 and cohesive failures in group 2 and 4.

**Conclusions:**

Fiber reinforced composites have higher compressive strength which can be used as an treatment option for incisal edge fractures.

** Key words:**Compressive strength, Failure modes, Fibers, Nanocomposites, Stereomicroscope, Universal testing machine.

## Introduction

Traumatic dental injuries have emerged as a significant health concern due to their high incidence as well as influence they exert on a person’s day-to-day activities regarding form and function (1). Fractures of dental crowns have been reported to constitute as much as 92% of all traumatic injuries to permanent teeth (2). Permanent incisor coronal fractures account for 18-22% of all dental hard tissue damage with simple cases involving enamel and dentin making up 28-44% and complex cases involving enamel, dentin, and pulp accounting for 11–15%. Of those, maxillary incisors are implicated in 96% (2). Owing to protrusion and growth mechanism of maxillary anteriors they are more susceptible to trauma and crown fracture (3,4).

Numerous techniques have been recorded for restoring a fractured incisal edge to original shape and color (5). A widely recognized treatment is the reattachment of the fractured incisal part (6). Some studies found in-vitro fracture resistance of reattached incisor fragments similar to intact teeth whereas other studies found that it was less effective than resin composite buildups (7). The goal is to maintain healthy tooth structure through a minimally invasive treatment approach (3). Conservative preparations are possible since adhesive restorative materials adhere well to tooth structure but their results were questionable (8). To enhance their physico-mechanical properties, various types of fibers were incorporated into the resin-matrix such as carbon, glass, vectran, kevlar and polyethylene fibers (9,10).

Glass Fibre reinforced Composites (FRCs) offers numerous advantages, including non-corrosiveness, high toughness, biocompatible, making it suiTable for dental applications (11,12). EverStick C&B incorporates 4000 continuous silanized E-glass fibers, combined with a light-polymerizable semi-interpenetrating polymer network (semi-IPN) of PMMA and Bis-GMA resin, enhancing its clinical utility and aesthetic appeal (13).

The efficacy of fiber reinforcement relies on several variables such as type of resins utilized, proportion of fibers in resin matrix, fiber length, fiber form, fiber orientation, adhesion between fibers and polymer matrix and saturation of fibers with resin (14,15,16).

Universal testing machine (UTM) measures fracture resistance of materials while testing their compressive and tensile strengths (17). Mode of bond failure like cohesive, adhesive and mixed were examined under stereomicroscope (18).

Although there is a solid understanding of FRCs as a material, their application as reinforcement for restorative composite resins in the treatment of anterior tooth fractures remains less investigated. The current study aims to compare the compressive strengths of two nanofill composites with and without fiber reinforcement to treat incisal edge fractures.

## Material and Methods

The inclusion criteria consist of extracted human maxillary central incisors with intact incisal edges. Teeth showing caries, visible coronal fractures, attrition were excluded. Sample size determination using G* power software (Version:3.1.9.2), with effect size of 0.45, power of study 80%, type-I α error 0.05 resulted in sample size of 60. Sixty freshly extracted human maxillary central incisors are collected cleaned of tissue debris, calculus using an ultrasonic scaler, and stored in a 0.1% thymol solution until use. A silicone key was used as a guide for restoring the tooth crown. Each tooth was prepared obliquely, with a 4mm incisal edge and a 6mm mesiodistal width using a diamond disc. The samples (n=60) were divided into four groups (n=15) based on the presence or absence of fiber reinforced composite restoration.

Group I: Nanofill composite (Filtek Z350XT) without fiber reinforcement: The fracture line was beveled to a 2mm width circumferentially at a 45° angle using a TC-S21 diamond bur (Mani Inc., Japan). The prepared tooth surface was etched with 37% phosphoric acid gel for 15 seconds, rinsed, and air-dried (3,19,20). Two coats of bonding agent (Single Bond Universal, 3M, US) are applied, followed by air thining for 5 seconds from distance of 1cm from the surface and light-cured for 20 seconds (21). The lost tooth structure was incrementally restored with nanofill composite (Filtek Z350XT, 3M, US), each layer polymerized for 30 seconds, plus an additional 20 seconds from labial and lingual sides. The crown length is restored to the original level using a silicone key (3,4,5,20).

GROUP II: Nanofill composite (Filtek Z350XT) with fiber reinforcement: The fracture line was beveled to a 2mm width circumferentially at a 45° angle using a TC-S21 diamond bur (Mani Inc., Japan), with a 5mm-long, 2mm-wide mesiodistal groove created on the incisal edge and three 2mm-long, 1mm-wide labiopalatal grooves spaced 1mm apart using a round diamond abrasive (BR-45(1mm diameter) Mani Inc., Japan). The etched tooth surface received two coats of bonding agent followed by air thining for 5 seconds from distance of 1cm from the surface and light-cured for 20 seconds (21). A layer of flowable composite (3M Filtek Supreme Flow, US) was applied, and a 2mm wide glass fiber (GC Everstick, Japan), was embedded into a 2mm prepared groove extending 5mm mesiodistally and three 1mm wide fibres were placed extending 2mm labiopalatally parallel to each other above the reduced incisal edge, and light-cured for 20 seconds. The remaining portion of the incisal edge was incrementally built with nanofill composite (Filtek Z350XT, 3M, US), with each increment polymerized for 30 seconds, followed by an additional 20 seconds of polymerization from both the labial and lingual sides. A silicone key was used to restore crown length to the original level (3,4,5,20).

GROUP III: Nanofill composite (GC Solare Sculpt) without fiber reinforcement: A similar protocol to group 1 was followed, using bonding agent (G-Premio Bond Universal, Japan) and nanofill composite (GC Solare Sculpt, Japan).

GROUP IV: Nanofill composite (GC Solare Sculpt) with fiber reinforcement: A similar protocol to group 2 was followed using bonding agent (G-Premio Bond Universal, Japan), flowable composite (G-Aenial Universal Injectable, Japan) and nanofill composite (GC Solare Sculpt, Japan).

**Figure 1:**
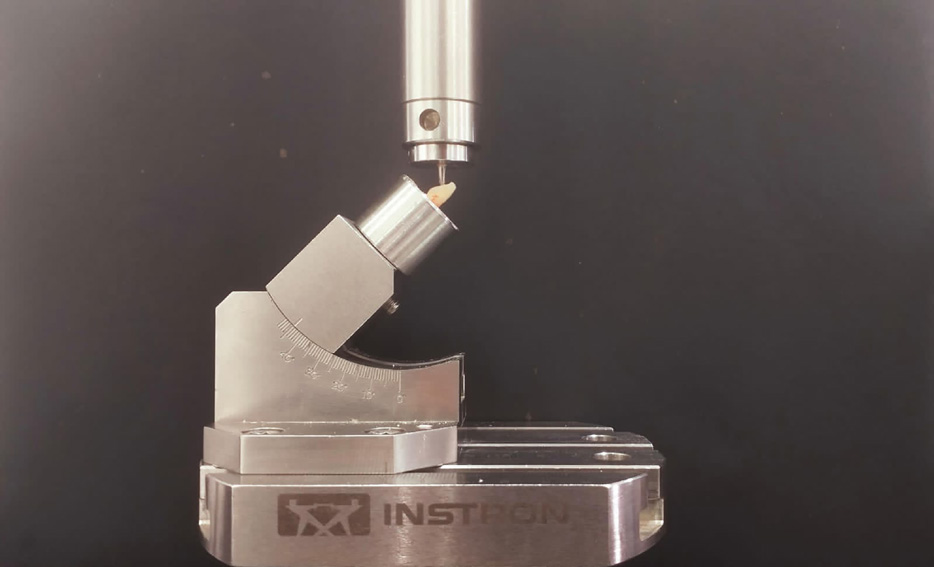
Compressive strength testing using universal testing machine.

Samples were subjected to fracture resistance using a Universal testing machine (UTM) (Instron E 3000, USA) as shown in Figure 1. A 2mm-diameter spherical steel jig, mounted on a UTM, transmitted loads to teeth at a cross-head speed of 1 mm/min, with each tooth oriented at a 45° angle (19). Maximum force applied during testing was recorded in Newtons (N). Fracture patterns were evaluated using Stereomicroscope (Olympus Pvt.Ltd.,India) at 30X (8,20). Each sample was grouped based on one of three failure modes: adhesive failure, cohesive failure or mixed failure. The obtained data was statistically analysed using one-way Analysis of variance (ANOVA) and Tukey’s post-hoc test.

## Results

The compressive strength of four groups was compared using one-way ANOVA, followed by Tukey’s post-hoc test for multiple pairwise comparisons. One-way ANOVA revealed statistically significant differences among the four groups, with the highest mean compressive strength observed in Group 2 (1369.40N) and Group 4 (1353.65N), and the lowest in Group 3 (290.45N) ( Table 1). Tukey’s post-hoc test indicated significant differences, with Group 2 showing higher compressive strength than Group 1, and Group 4 also exhibiting significantly greater strength than Group 1. Similarly, Group 2 and 4 demonstrated significantly higher strength than Group 3. However, no significant differences were noted between Group 1 and Group 3 or between Group 2 and Group 4 ( Table 2). Regarding failure modes cohesive failure was most prevalent in Groups 2 and 4, while mixed failure dominated in Groups 1 and 3 ( Table 3).

## Discussion

Restoring traumatized teeth while achieving optimal aesthetics, form and function has long posed a clinical challenge (22). Clinicians must carefully consider treatment plans that are minimally invasive with comprehensive restoration (23). One such approach incorporates fibers into the restorative resins, facilitating enhanced retention and minimizing the risk of clinical failures (22).

Filtek Z350XT nanofill composite with fiber reinforcement showed the highest compressive strength in study groups. This improvement is likely due to the synergistic effect of fiber-reinforced composites (FRC) with composite materials, which enhances mechanical performance by distributing forces over a larger surface area (24,25). Luthria *et al*.,(26) reported that fracture resistance significantly improves when composite resin is reinforced with impregnated glass fibers. The superior performance observed in the FRC group may be attributed to the fiber length (5 mm mesiodistally and 2 mm labiopalatally) used in this study, aligning with Petersen *et al*.’s findings that stress is transferred effectively when fiber length meets or exceeds the critical length of 0.5–1.6 mm (27). Other factors positively affecting fracture resistance in this study include the fiber bundle width (2 mm), thickness (1.15 mm) with 4,000 glass fibers, and the number of fibers on the incisal edge. One mesiodistal and three labiopalatal grooves were created on the incisal edge, which might have enhanced fracture resistance, as grooves can absorb surface stresses, as noted by AD Loguercio *et al*. (28). The enhanced fracture resistance of FRC combined with Filtek Z350XT composite in this study align with findings by Upadhyay RK *et al*., who attributed Filtek Z350XT’s high compressive strength to its nanofillers, which increase filler volume and material strength (29). This also corresponds with Lippo Lassila’s research, which demonstrated that the semi-IPN polymer matrix and specific fiber properties in EverStick C&B glass fibers improve bonding performance (15).

The use of Single Bond Universal (SU) bonding agent employed in FRC with Filtek Z350XT composite likely contributed to the improved outcomes. SU contains 10-MDP (10-methacryloyloxydecyl dihydrogen phosphate) and Vitrebond copolymer, which enables chemical bonding with dentin through ionic bonds (30,31). Mami K *et al*. reported that Vitrebond copolymer interacts with calcium ions in dentin’s hydroxyapatite, enhancing bond durability (32).

Beveling the enamel margin at a 45° angle and extending 2 mm beyond the fractured incisal edge likely increased compressive strength. This aligns with findings by JB Black *et al*. and Jamshid Bagheri *et al*., who found that a 45° bevel with a 2 mm width improves restoration retention and offers more accessible cavosurface angles than a butt joint (33,34).

FRC combined with Solare Sculpt nanofill composite showed the second-highest compressive strength. This improved fracture resistance may be due to the strategic factors like fiber length, thickness, number and the grooves prepared likely influenced the mean fracture resistance values. In groups 2 and 4, a flowable composite was used to bond the fibers. Davari *et al*. found that it masked the junction between tooth and composite, restoring up to 35% of fracture resistance in intact teeth (35). On the other hand Tezvergil *et al*. observed no impact from its use (18).

Groups 1 and 3, without fibers and grooves, showed the lowest strength, underscoring the role of fibers and grooves in reinforcing restoration. The fibers in FRC enhance structural integrity, especially under stress, while grooves improve retention by securely anchoring the restoration. Without grooves, the mechanical interlocking is weaker, increasing the risk of dislodgement or debonding, which may explain the lower strength observed in groups 1 and 3.

Group 2 exhibited significantly higher mean compressive strength than Group 1 and 3, likely due to the incorporation of GFRC and grooves for increased mechanical retention. These findings align with studies by Garoushi *et al*. and Belli *et al*., which reported higher fracture loads in FRC groups (36,37). This contrasts with findings by C.L. Pereira *et al*., who reported no increase in flexural strength with fiber-composite laminates due to limitations like small specimen size and the use of only a small and single fiber strip (38).

Group 4 showed significantly higher mean compressive strength than Group 1and 3. This supports findings by Garoushi, Vallitu, Belli, and Tezvergil, who reported that FRC substructures enhance load-bearing capacity compared to particle filled composites (PFC) alone (18,36,37,39,40). Geerts *et al*. found glass FRC to be the best reinforcement for PFC in aesthetic, space-limited cases (41). In contrast, Hamza *et al*. observed no significant difference in flexural strength and fracture toughness between glass and polyethylene fiber reinforcements (42).

SU was used in Groups 1 and 2, while G-Premio Bond (GB) was used in Groups 3 and 4. GB’s higher water content (25%) compared to SU (10-15%) may explain its lower bond fatigue resistance, as increased water can weaken the adhesive layer (43). Yamauchi K(44) noted that residual water in GB could cause cracks at the GB-dentin interface. GB’s composition includes 10-MDP, 4-MET(4-methacryloxyethyl trimellitic acid), and 10-MDTP(10-methacryloyloxydecyl dihydrogen thiophosphate), whereas SU contains 10-MDP and unique polyalkenoic acid copolymer, which enhances dentin adhesion (30,31). These compositional differences likely account for lower bond strength in GB groups (3 and 4) compared to SU groups (1 and 2).

Group 1 predominantly showed mixed failures with some adhesive failures, while Group 3 exhibited more mixed failures with cohesive failures. FRC restorations primarily failed cohesively, followed by mixed failures, likely due to FRC’s high load-bearing strength. This aligns with Garoushi *et al*., (36) who found cohesive fractures common in glass FRCs, as well as by PS Praveen Kumar and Talat *et al*., (24,25) where stereomicroscopic analysis showed 10% adhesive and 70% cohesive failures in glass FRC specimens.

Fracture resistance varies across FRC studies due to differing techniques and materials. PS Praveen Kumar *et al*., (24,25) and Gayathri *et al*., (3) reported 830-860 N with a 1mm bevel, while Chandra Sekhar *et al*., (20) and Patnana *et al*., (10) observed lower values(434.87 N and 218.57 N) with other fiber types and designs. Talat’s study reported a range of 794-803 N with glass FRC and 1mm bevel used (22). In this study Group 2 and Group 4 showed higher mean fracture resistance (1369.40 N and 1353.65 N) likely due to factors like groove preparation, fiber placement, and composite type, which together enhanced outcomes.

This study has several limitations that may affect its clinical applicability. As an *in vitro* study, it does not fully replicate oral conditions, such as the presence of saliva, variations in pH, or long-term wear, all of which can influence restoration performance. Exploring effects of different types and orientations of fibers, variations in preparation techniques could provide further insights into optimizing the performance of FRC in restorative dentistry. Further clinical studies with diverse setups are needed to validate and broaden these findings for practical use in restorative dentistry. Influence of groove prepared on sensitivity of tooth and pulpal health are not evaluated.

## Conclusions

Based on the study’s findings and limitations, following conclusions can be drawn: The addition of fiber reinforcement significantly improved the compressive strength of both composites, suggesting that fibers enhance the mechanical properties of composite restorations for greater durability. Failure modes varied across groups; composites without fiber reinforcement predominantly experienced mixed failures, while those with fiber reinforcement mainly exhibited cohesive failures, indicating improved fracture resistance. This indicates that the presence of fibers and grooves may influence the fracture behaviour of the restorations, leading to different failure patterns.

## Figures and Tables

**Table 1:** Summary of compressive strength (N) in four groups (1,2,3,4).

Group	Mean	SD	SE	95% CI for mean	
				Lower	Upper
Group 1	428.93	182.93	47.23	327.63	530.24
Group 2	1369.40	258.30	66.69	1226.36	1512.45
Group 3	290.45	131.60	33.98	217.57	363.32
Group 4	1353.65	342.46	88.42	1164.00	1543.30

**Table 2:** Compressive strength (N) Pair-wise comparison of four groups (1,2,3,4) by Tukey’s multiple post-hoc procedure.

Group	Group 1	Group 2	Group 3	Group 4
Mean	428.93	1369.40	290.45	1353.65
SD	182.93	258.30	131.60	342.46
Group 1	-	P = .0002*	P = .4065	P = .0002*
Group 2	P = .0002*	-	p= .0002*	P = .9980
Group 3	P = .4065	P = .0002*	-	P = .0002*
Group 4	P = .0002*	P = .9980	P = .0002*	-

*P<.05

**Table 3:** Modes of failure comparison of four groups (1,2,3,4).

Groups	Modes of failure						
	Adhesive	%	Cohesive	%	Mixed	%	Total
Group 1	4	26.67	2	13.33	9	60.00	15
Group 2	1	6.67	11	73.33	3	20.00	15
Group 3	3	20.00	4	26.67	8	53.33	15
Group 4	1	6.67	9	60.00	5	33.33	15
Total	9	15.00	26	43.33	25	41.67	60

Chi-square=14.7940, *P*= .0220* *P<.05

## Data Availability

The datasets used and/or analyzed during the current study are available from the corresponding author.
